# Dietary Supplementation of* Phoenix dactylifera* Seeds Enhances Performance, Immune Response, and Antioxidant Status in Broilers

**DOI:** 10.1155/2016/5454963

**Published:** 2016-11-28

**Authors:** Ali H. El-Far, Hamada A. Ahmed, Hazem M. Shaheen

**Affiliations:** ^1^Department of Biochemistry, Damanhour University, Damanhour, Egypt; ^2^Department of Nutrition and Veterinary Clinical Nutrition, Damanhour University, Damanhour, Egypt; ^3^Department of Pharmacology, Damanhour University, Damanhour, Egypt

## Abstract

The date palm (*Phoenix dactylifera*) seeds were utilized in some traditional medical remedies and have been investigated for their possible health benefits. This proposed study wanted to assess the effect of date palm seeds (DPS) dietary supplementation in comparison to mannan-oligosaccharides (Bio-Mos®) and *β*-glucan over antioxidant and immunity events that have effect on growth and carcass performances of broilers. An aggregate of 180, one-day-old, chicks were raised in the wire-floored cages and allotted into control, Bio-Mos (0.1%  Bio-Mos), *β*-glucan (0.1%  *β*-glucan), DPS2 (2% date crushed seeds), DPS4 (4% date crushed seeds), and DPS6 (6% date crushed seeds) groups. Broilers in DPS2 and DPS4 groups showed significant variations (*P* < 0.05) in relative growth rate (RGR), feed conversion ratio (FCR), and efficiency of energy utilization in comparison to control group. Moreover, all DPS fed groups showed significant increases (*P* < 0.05) in serum reduced glutathione (GSH) values. Meanwhile, both serum interferon-gamma (IFN-*γ*) and interleukin-2 (IL-2) levels were significantly increased (*P* < 0.05) in DPS2. Consequently, obtained data revealed a substantial enhancement of performance, immunity, and antioxidant status by DPS supplementation in broiler that might be related to the antioxidant and immune-stimulant constituents of* P. dactylifera* seeds.

## 1. Introduction

Wide usage of antimicrobial agents has added to an imbalance among pathogenic and ordinary intestinal microflora as well as the development of multiple antibacterial resistance cases. In the poultry industry, natural feed additives have got the potential of reduction of poultry enteric diseases [1, 2]. Both mannan-oligosaccharides and *β*-glucan are extracted from* Saccharomyces cerevisiae *[3] and both were used as a feed additive in many poultry farms to enhance poultry execution and lower the liability to the pathogens propagation of the gastrointestinal tract and respiratory system [4, 5]. *β*-Glucan is a glucose polymer as basic component in the yeast and fungi that enhances defenses against bacterial challenge and increases the growth performance [6].

*Phoenix dactylifera* is a major source of nutrients for mankind [7]. The date palm seeds (DPS) are also called pips, stones, kernels, or pits that represent about 6–12% of whole date [8]. DPS are rich in minerals and vitamins. The chemical composition of DPS varies according to the nature of cultivating land. As far as dry weight, the chemical components of DPS have contained about 5–10% of moisture, 5–7% of protein, 7–10% of oil, 10–20% of crude fiber, 55–65% of carbohydrates, and 1-2% of ash [9]. The antioxidant effect of DPS regards its phenolic compounds of anticarcinogenic and anti-inflammatory activities [10].

Using medicinal herbs to decrease the participation of chemicals through the worldwide tendency to return to the natural supplements has been supported by the World Health Organization [11]. Many studies were done to investigate the usage of the medicinal plant as feed additives such as basil supplementation and/or chamomile that improves the immunity and performance of broiler [12] and* P. dactylifera* in New Zealand rabbits [13].* P. dactylifera* is a prevalent diet among the Middle Eastern populations; its fruits are framed of a fleshy pericarp and seed, constituting between 10% and 15% of date fruit weight [14].* P. dactylifera* has been developed in the Middle East over no less than 6000 years ago [15].

This trial was designed to compare the possible effects of mannan-oligosaccharides, *β*-glucan, and different levels of* P. dactylifera* seeds (DPS) on performance, and carcass characteristics, beside the oxidative and immunity events in broilers.

## 2. Materials and Methods

### 2.1. Chemical Analysis of* Phoenix dactylifera* Seeds

*P. dactylifera* seeds were collected at tamr stage from Al-Beheira Governorate, Egypt, and kept at 4°C. DPS were crushed to produce a fine powder subsequently analyzed [16] for the dry matter at 105°C for 3 hours in a hot air oven. Crude protein was determined by Kjeldahl method using Gerhardt Vapodest 10 and Gerhardt Turbotherm and lipid by ether extraction (Gerhardt Soxtherm). Fibers were determined by extraction with 0.5 M H_2_SO_4_ and 0.5 M NaOH (Gerhardt Fibertherm), drying, and ashing, after which ash substance was resolved after burning in a furnace at 550°C for 12 hours. Moreover, lysine and methionine were determined by calculation [17].

### 2.2. *Phoenix dactylifera* Seeds Extract

The crushed* P. dactylifera* seeds were extracted with methanol [18]. Quickly, 15 g of* P. dactylifera* seeds powder was extracted with 100 mL of methanol for 24 hours with occasional shaking. The extract was filtered and evaporated to dryness in a vacuum.

### 2.3. Gas Chromatography-Mass Spectrometry (GC-MS) Analysis

The chemical components of* P. dactylifera* seeds were carried on using Trace GC Ultra-ISQ mass spectrometer with a direct capillary column TG-5MS with 30 m × 0.25 mm × 0.25 *μ*m film thickness. The oven temperature was adjusted at 60°C and then raised by 5°C/minute to 280°C. The temperatures of both injector and detector were adjusted at 250°C. Helium was used as a carrier gas at a constant flow rate of 1 mL/minute for 21.03 minutes. The solvent retention time was 2 minutes and the diluted samples of 1 *μ*L were injected by using autosampler AS3000 in the splitless mode. The segments were recognized by examination of their delay times and mass spectra with those of NIST 11 mass spectral database [19].

### 2.4. Birds and Dietary Treatments

This work was completed at the Faculty of Veterinary Medicine, Damanhour University, to evaluate the possible effects of Bio-Mos (mannan-oligosaccharides, produced by Alltech Co., USA), *β*-glucan ((1,3) *β*-D-glucan) which is produced by Beta-Mune™, Germany, and different levels of DPS supplementations on growth profile, carcass attributes, oxidative status, and immune events in broilers. One hundred eighty of one-day-old Ross 308 chicks with a mean body weight of 39.50 g were acquired from the local broiler chicken hatchery and then randomly allocated into six experimental groups (three replicates each of ten birds).

The study convention was affirmed by the Committee on the Ethics of Animal Experiments of Alexandria University, Egypt. The birds were raised in wire-floored cages and fed on a starter diet from the beginning of the experiment till the 3rd week of age, followed by a finishing diet to the end of experiment. The chicks were allocated into control (received the basal eating program), Bio-Mos (received the basal diet containing 0.1% Bio-Mos), *β*-glucan (got the basal eating routine containing 0.1%  *β*-glucan), DPS2 (received diet containing 2% date crushed seeds), DPS4 (received diet containing 4% date crushed seeds), and DPS6 (received diet containing 6% date crushed seeds) groups from 1st to 42nd days of age.

The incubation temperature of 32°C was gradually decreased until reaching 26°C by the third week of age. The chicks were exposed to a 23-hour light regimen. Both components and synthetic materials of the basal diets are showed in Tables 1 and 2. The basal diets were mixed using National Research Council instruction [20] where protein percentages are 22.6 and 18.14 g/100 g for starter and finisher diets, respectively. Birds were vaccinated as follows: Clone Ma5 by eye drop on the 7th day of age, Gumboro Intermediate Plus (Bursine Plus vaccine) eye drop on the 14th day, LaSota vaccine eye drop on the 18th day of age, and finally LaSota vaccine plus IBD vaccine eye drop on the 28th day.

### 2.5. Evaluation of Growth Performance

Body mass development and intake of feed in the treatment groups were weekly recorded. The weight gain in grams was calculated as the difference between two consecutive body weights. In addition, feed conversion ratio, relative growth rate (RGR), and efficiency of energy utilization were also calculated.

### 2.6. Hemagglutination Inhibition (HI) Test

Three categories of blood specimens were taken from the birds of each experimental group on the 14th, 21st, and 42nd days of age. Blood specimens were taken for collection of sera to examine the antibodies titer against Newcastle disease vaccine, using the HI test as an indicator of the bird's immune health in the different experimental classes. Microtechnique to HI test was carried out following [21], while geometric mean titer (GMT) was measured after [22].

### 2.7. Carcass Properties

After 6 weeks of the experiment, five chickens per class were randomly chosen, fasted for 12 hours, and then weighed after which they were sacrificed and weighed to determine the dressing percentage, whereas liver, spleen, thymus, and bursa were weighed and the relative weights of chickens to their body mass were calculated. Gizzards, heart, and visible fat, from each chick, were also weighed.

### 2.8. Biochemical Analysis

Blood samplings were gathered from the wing vein on the 21st and 42nd days of the trial. Centrifugation of blood at 3000 rpm for 5 minutes to harvest clear sera was done. The gathered sera were exposed to biochemical investigations of reduced glutathione (GSH) levels [23]. Meanwhile, the interferon-gamma (IFN-*γ*) and interleukin-2 (IL-2) levels were determined by ELISA kits that were purchased from Cusabio Company, while nitric oxide (NO) ELISA kit was purchased from WKEA Company ELISA kits. The UNICO 2100UV-Spectrophotometers, ELx800 Absorbance Microplate Reader, and other lab hardware help were utilized as a part of biochemical examinations.

### 2.9. Statistical Analysis

All values were stated as means ± SD. The statistical measures were handled by the SPSS programming (SPSS 22). One-way ANOVA was used followed by Duncan's multiple range tests, when the impact was significant, in order to separate the significant contrasts between dietary applications. All declarations of significance were depending on *P* < 0.05.

## 3. Results and Discussion

**Figure 1:**
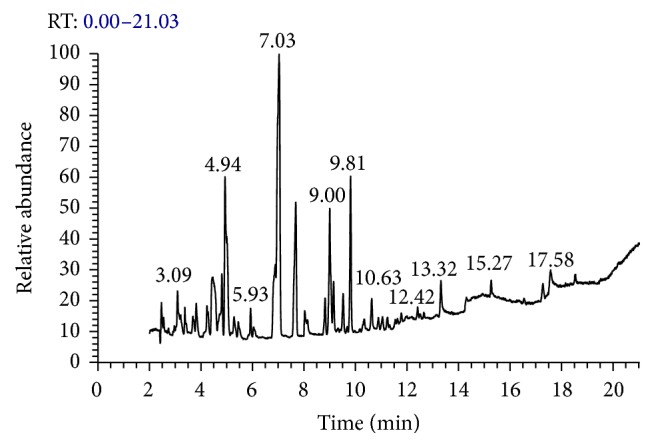
GC-MS chromatogram of* P. dactylifera* seeds methanolic extract.

The data presented in Figure 1 and illustrated in Table 3 explore 15 major different components present in* P. dactylifera* seeds that were analyzed by GC-MS. The obtained data identified the presence of some antioxidant and immune-stimulant compounds such as 4,6-dimethyl-3-(4-methoxyphenyl) coumarin (14.73%), 4-methylcinnamic acid (11.44%), 6-hydroxy-7-methoxycoumarin (8.71%), and 7-allyloxy-4-methylcoumarin (2.20%). Cinnamic acid and its derivatives were identified in* P. dactylifera* seeds of different date varieties [24–27]. More than 1300 coumarins have been documented from plant seeds, roots, and leaves possess an antioxidant, anticancer, anti-inflammatory, and antimicrobial properties [28–32].

The mean body weights of broiler nourished on basal diets and Bio-Mos and *β*-glucan supplemented diets compared with broiler chicks fed on diets that contained DPS at 2, 4, and 6% were illustrated in Table 4 that revealed higher significant increases in body weights and weight gain of broilers fed diets containing Bio-Mos, *β*-glucan, and DPS at levels of 2% and 4% when compared with the control one. Also, it was noticed that there were significant differences in relative growth rate (RGR) in all treated groups except those of DPS6 group when compared with the control one (Table 4); the highest RGR was observed in Bio-Mos, *β*-glucan, DPS2, and DPS4 groups (192.91 ± 0.13, 192.90 ± 0.07, 193.19 ± 0.07, and 193.06 ± 0.09, resp.). In regard to the feed intake, it was increased in all treated broiler groups in comparison to those of the control one (Table 4). Concerning total feed conversion ratio, chicks of DPS2 and DPS4 groups recorded the best feed conversion ratio in comparison with those of control one (1.58 ± 0.03 and 1.60 ± 0.03 versus 1.75 ± 0.04, resp.) and achieved the best energy utilization efficiency (4.94 ± 0.10 and 5.02 ± 0.10 versus 5.46 ± 0.14, resp.).

The increase in body weight and higher body weight gain due to the presence of Bio-Mos and *β*-glucan agree with those of [5, 33] which concluded the effect of mannan-oligosaccharides and *β*-glucans supplementation shows significant increase in body weight gain and enhancement in the feed efficiency in relation to the control diet. This advance may be with regard to the improvement of intestinal mucosal integrity and the increase in the absorption and utilization of the dietary nutrients [34].

DPS containing diets at levels of 2% and 4% showed higher significant increases in body weights and weight gain in broilers. The research study on DPS conveys the increment in the body weight due to DPS to mannan-oligosaccharides that were already detected in DPS [35] in addition to selenium, phenolic, and carotenoid compounds of DPS [36].

The effects of diets containing Bio-Mos and *β*-glucan comparing with different levels of DPS at 2, 4, and 6% on antibody level against Newcastle disease virus (NDV) of broiler chickens during 14th, 21st, and 42nd day of age in relation to control group were presented in Table 5. On the 14th day of age, there were insignificant differences among different treated groups. Meanwhile, on the 21st day, the birds fed diets containing DP at levels 6 and 4% showed a significant antibody titer against NDV, respectively, when compared with control one; also on the 42nd day the DPS2, DPS4, and DPS6 showed a significant antibody titer against NDV.

The data presented in Table 6 stated the carcass characteristics of control and treated birds. The dressing percentages in different experimental groups showed significant improvement in dressing percentage when compared with control one except those of DPS6 group. Conversely, the liver, heart, and gizzard weights showed no significant difference among different experimental groups. Table 6 also shows an enhancement in immune organ weight; spleen weight showed a numerical increase in treated groups. Also, thymus weights showed significant increases in DPS2 and DPS4 groups. The increment in antioxidant status of animals improves their growth performance, production, and reproduction [37]. Because mannan-oligosaccharides are not digested, they stimulate the lymphatic system of the gastrointestinal tract and general immunity [38].

The data belonging to the biochemical study were illustrated in Tables 7 and 8. The serum levels of GSH were significantly increased (*P* < 0.05) in DPS treated group, especially at 21st day. Indeed, its levels in Bio-Mos and *β*-glucan treated groups were significantly increased (*P* < 0.05) at 42nd day of the experiment. In regard to serum NO, its levels were unchanged at 21st day and slightly decreased in all treated groups in comparison to control one (Table 7). The antioxidant activity of Bio-Mos, *β*-glucan, and DPS treated group is evidenced by the significant increase in the serum levels of GSH. The obtained data reported a talented antioxidant effective feed additive in broiler diets through DPS besides yeast cell wall prebiotics [39]. The methanolic extract of DPS is considered as an antioxidant source of *β*-carotene and phenolic compounds [40]. This antioxidant effect became proportional to the phenolic contents [41]. The significant decrease in MDA level in testicular tissue of DPS treated male rats in comparison with control one may be attributed to the antioxidant effect of* p*-coumaric acid, ferulic acid, flavonoids, sinapic acids, and procyanidins [42].

The data of cellular immunity were obtained in Table 8 in which both IFN-*γ* and IL-2 were significantly increased (*P* < 0.05) in all DPS treated groups. In DPS2 group the highest levels of IFN-*γ* and IL-2 were at 42nd day while being at 21st day in Bio-Mos group. In regard to the comparison between the different concentrations of date seeds supplementations, the DPS2 group improved the antioxidant and cellular immunity in treating chicks. Dietary antioxidants exert their positive effects on the elimination of reactive oxygen species and subsequently prevent the activation of the inflammation process [43]. With regard to the serum production of cytokines, which were used for further understanding of immune status, we observed an upregulation of IL-2 and IFN-*γ* in broiler chickens in the Bio-Mos, *β*-glucan, and DPS groups comparable to the control one, in which IFN-*γ* is a soluble cytokine that is the only member of type II class of interferon known as immune interferon [44]. In addition, IL-2 is a type of cytokine that regulates the activities of leukocytes, often lymphocytes that are accountable for immunity [45]. In addition, IFN-*γ* production was augmented due to *β*-glucan in poultry [46, 47].

## 4. Conclusion

From the obtained data, we can conclude that the supplementation of broilers with ration containing DPS at levels 2% and 4% is of great beneficial improvements in broiler health producing healthy birds with higher body weight, despite enhancements of body weight, gain, organ weight, antibody titer, IFN-*γ*, IL-2, and antioxidant status in comparison to mannan-oligosaccharides and *β*-glucan.

## Figures and Tables

**Table 1:** Ingredients percentages and calculated composition analysis of the experimental starter diets (as fed basis).

Ingredients	Basal diet	DPS2	DPS4	DPS6
Corn	55	53.09	51.17	49.27
SBM (CP 44%)	32.04	32	32	32
Corn gluten (CP 62%)	5.5	5.6	5.66	5.7
Corn oil	3.5	3.39	3.27	3.17
Limestone	1.35	1.33	1.31	1.28
Dicalcium phosphate	1.74	1.74	1.74	1.74
L-lysine^*∗*^	0.28	0.28	0.28	0.27
Dl-methionine^*∗∗*^	0.14	0.12	0.12	0.12
*P. dactylifera* seeds	0	2	4	6
Vitamin and mineral premix^*∗∗∗*^	0.3	0.3	0.3	0.3
NaCl	0.15	0.15	0.15	0.15
Total	100	100	100	100
Estimated and analyzed composition				
ME	3069.59	3070.39	3069.38	3070.05
CP %	22.6	22.6	22.59	22.58
Lysine %	1.34	1.34	1.34	1.34
Methionine %	0.5	0.5	0.5	0.5
Calcium %	1	1	1	1
Av. (P) %	0.45	0.45	0.45	0.45

SBM: soybean meal. ME: metabolizable energy (Kcal/kg diet). CP: crude protein. Av. (P): available phosphorous. ^*∗*^L-lysine, 99% feed grade. ^*∗∗*^Dl-methionine, 99% feed grade, China. ^*∗∗∗*^Vitamin and mineral premix (Hero mix) produced by Hero pharm Co., Egypt.

**Table 2:** Ingredients percentages and calculated composition analysis of the experimental finisher diets (as fed basis).

Ingredients %	Basal diet	DPS2	DPS4	DPS6
Corn	67.58	65.54	63.64	61.55
SBOM (CP 44%)	23	23.09	23.20	23.35
Corn gluten (CP 62%)	3.0	3.0	3.0	3.0
Corn oil	3.4	3.36	3.27	3.17
Limestone	1.15	1.12	1.10	1.05
Dicalcium phosphate	1.27	1.30	1.30	1.30
L-lysine^*∗*^	0.14	0.13	0.13	0.12
Dl-methionine^*∗∗*^	0.01	0.01	0.01	0.01
*P. dactylifera* seeds	0.0	2.0	4.0	6.0
Vitamin and mineral premix^*∗∗∗*^	0.3	0.3	0.3	0.3
NaCl	0.15	0.15	0.15	0.15
Total	100	100	100	100
Calculated and analyzed composition				
ME	3187.63	3189.41	3188.58	3188.10
CP %	18.14	18.13	18.12	18.14
Lysine %	0.96	0.96	0.96	0.96
Methionine %	0.32	0.32	0.32	0.32
Calcium %	0.80	0.80	0.80	0.80
Av. (P)	0.35	0.35	0.35	0.35

SBM: soybean meal. ME: metabolizable energy (Kcal/ kg diet). CP: crude protein. Av. (P): available phosphorous. ^*∗*^L-lysine, 99% feed grade. ^*∗∗*^Dl-methionine, 99% feed grade, China. ^*∗∗∗*^Vitamin and mineral premix (Hero mix) produced by Hero pharm Co., Egypt.

**Table 3:** GC-MS analysis of *P. dactylifera* seeds.

Peak	Retention time (minutes)	Name	Area%	Molecular weight	Molecular formula
1	2.47	1-Ethynyl-3,trans(1,1-dimethylethyl)-4,cis-methoxycyclohexan-1-ol	1.83	210	C_13_H_22_O_2_
2	3.09	Ethanol, 2-ethoxy-	2.77	90	C_4_H_10_O_2_
3	3.82	Pentane, 3-ethyl-2,4-dimethyl-	2.14	128	C_9_H_2_O_2_
4	4.45	*α*-Aminobutyric acid	8.98	103	C_4_H_9_NO_2_
5	4.82	sec-Butyl acetate	4.48	116	C_6_H_12_O_2_
6	4.94	4,6-Dimethyl-3-(4-methoxyphenyl) coumarin	14.73	280	C_18_H_16_O_3_
7	7.04	cis-5,8,11,14-Eicosatetraenoic acid	25.64	304	C_20_H_32_O_2_
8	7.68	4-Methylcinnamic acid	11.44	162	C_10_H_10_O_2_
9	8.82	7-Allyloxy-4-methylcoumarin	2.20	216	C_13_H_12_O_3_
10	9.00	Minoxidil	8.20	209	C_9_H_15_N_5_O
11	9.15	1-(N-Methylamino)-1-phenylpropane	2.05	149	C_10_H_15_N
12	9.52	2-Ethyl-2-(p-tolyl) malonamide	2.50	220	C_12_H_16_N_2_O_2_
13	9.81	6-Hydroxy-7-methoxycoumarin	8.71	192	C_10_H_8_O_4_
14	10.63	Mesitylene	1.91	120	C_9_H_12_
15	13.32	2H-Pyran, tetrahydro-2-(12-pentadecynyloxy)-	2.42	308	C_20_H_36_O_2_

**Table 4:** Growth performance parameters of control and treated groups.

Group	IWT	FWT	WG	FI	FCR	RGR	PER	EEU	Mort. rate %
Control	39.83 ± 0.55^a^	2073.7 ± 52.6^b^	2033.87 ± 52.09^b^	3501.07 ± 0.24^f^	1.75 ± 0.04^ab^	192.51 ± 0.10^c^	0.10 ± 0.001^a^	5.46 ± 0.14^ab^	8
Bio-Mos	39.91 ± 0.33^a^	2204.14 ± 54.56^a^	2164.23 ± 54.25^a^	3550 ± 0.27^a^	1.67 ± 0.05^bc^	192.91 ± 0.13^b^	0.03 ± 0.001^b^	5.20 ± 0.14^bc^	4
*β*-Glucan	40.08 ± 0.24^a^	2195.83 ± 34.26^a^	2155.75 ± 34.03^a^	3539.94 ± 0.28^b^	1.65 ± 0.03^bc^	192.9 ± 0.07^b^	0.03 ± 0.001^b^	5.16 ± 0.08^bc^	4
DPS2	39.96 ± 0.37^a^	2290.63 ± 44.46^a^	2250.67 ± 44.10^a^	3519.13 ± 0.23^e^	1.58 ± 0.03^c^	193.19 ± 0.07^a^	0.03 ± 0.001^b^	4.94 ± 0.10^c^	4
DPS4	40.08 ± 0.25^a^	2255.21 ± 40.60^a^	2215.13 ± 40.37^a^	3525.05 ± 0.20^c^	1.60 ± 0.03^c^	193.06 ± 0.09^ab^	0.03 ± 0.001^b^	5.02 ± 0.10^c^	12
DPS6	40.13 ± 0.25^a^	2023.96 ± 32.5^b^	1983.83 ± 32.27^b^	3520 ± 0.26^d^	1.78 ± 0.03^a^	192.31 ± 0.08^c^	0.03 ± 0.001^b^	5.59 ± 0.09^a^	4

Means within the same column carrying different letters are significantly different at *P* < 0.05.

Values are expressed as means ± SE.

IWT: initial body weight. FWT: final body weight. WG: weight gain. FI: feed intake. FCR: feed conversion ratio. RGR: relative growth rate. PER: protein efficiency ratio. EEU: efficiency of energy utilization.

**Table 5:** Hemagglutination inhibition (HI) titer of Newcastle disease virus in control and treated groups at 14th, 21st, and 42nd day of age.

Group	14th day	21st day	42nd day
Control	4.30 ± 0.06^a^	5.17 ± 0.19^c^	5.27 ± 0.15^b^
Bio-Mos	4.47 ± 0.03^a^	5.30 ± 0.15^bc^	5.47 ± 0.03^ab^
*β*-Glucan	4.43 ± 0.03^a^	5.20 ± 0.06^c^	5.43 ± 0.03^b^
DPS2	4.37 ± 0.09^a^	5.50 ± 0.06^abc^	5.73 ± 0.15^a^
DPS4	4.43 ± 0.03^a^	5.63 ± 0.03^ab^	5.67 ± 0.09^a^
DPS6	4.23 ± 0.07^a^	5.70 ± 0.10^a^	5.63 ± 0.07^a^

Means within the same column carrying different letters are significantly different at *P* < 0.05.

Values are expressed as means ± SE.

**Table 6:** Dressing percentage and relative weights for liver, heart, gizzard and spleen, and thymus and bursa of control and treated groups.

Item	Dressing %	Liver	Heart	Gizzard	Spleen	Thymus	Bursa
Control	68% ± 0.01^c^	40 ± 2.08^a^	9.83 ± 0.09^a^	31 ± 1.53^a^	1.7 ± 0.06^a^	2.83 ± 0.03^c^	2.10 ± 0.06^a^
Bio-Mos	71% ± 0.01^ab^	40.67 ± 1.76^a^	10.5 ± 0.29^a^	34.33 ± 0.67^a^	1.93 ± 0.07^a^	3.20 ± 0.06^a^	2.23 ± 0.07^a^
*β*-Glucan	72% ± 0.01^a^	42 ± 1.15^a^	10.67 ± 0.17^a^	34.33 ± 0.67^a^	1.9 ± 0.06^a^	3.23 ± 0.03^a^	2.23 ± 0.07^a^
DPS2	71% ± 0.01^ab^	41.67 ± 2.03^a^	10.33 ± 0.44^a^	32.67 ± 2.33^a^	1.83 ± 0.09^a^	3.13 ± 0.07^ab^	2.13 ± 0.09^a^
DPS4	71% ± 0.01^ab^	41.67 ± 2.03^a^	10.17 ± 0.6^a^	31 ± 0.58^a^	1.9 ± 0.06^a^	2.97 ± 0.09^ab^	2.20 ± 0.06^a^
DPS6	69% ± 0.01^bc^	40.33 ± 1.86^a^	10.43 ± 0.23^a^	32 ± 1.0^a^	1.77 ± 0.03^a^	3.03 ± 0.09^abc^	2.10 ± 0.06^a^

Means within the same column carrying different letters are significantly different at *P* < 0.05.

Values are expressed as means ± SE.

**Table 7:** The mean values of serum GSH and NO in control and treated groups.

	GSH (*µ*g/mL)		NO (*µ*mol/L)	
	21st day	42nd day	21st day	42nd day
Control	49.55 ± 3.29^b^	23.56 ± 4.71^b^	8.97 ± 0.04^a^	8.24 ± 0.27^bc^
Bio-Mos	22.31 ± 3.21^c^	45.97 ± 13.56^a^	8.09 ± 0.21^a^	7.68 ± 0.12^c^
*β*-Glucan	16.75 ± 1.86^d^	47.55 ± 13.59^a^	8.11 ± 0.62^a^	8.19 ± 0.21^b^
DPS2	67.44 ± 5.82^a^	34.65 ± 9.42^ab^	8.09 ± 0.32^a^	8.07 ± 0.04^ab^
DPS4	55.91 ± 2.67^ab^	31.71 ± 0.4^ab^	8.18 ± 0.55^a^	8.15 ± 0.14^a^
DPS6	55.46 ± 4.69^ab^	34.2 ± 1.74^ab^	8.17 ± 0.31^a^	8.00 ± 0.09^bc^

Means within the same column carrying different letters are significantly different at *P* < 0.05.

Values are expressed as means ± SE.

**Table 8:** The mean values of serum IFN-*γ* and IL-2 in control and treated groups.

	IFN-*γ* (pg/mL)		IL-2 (pg/mL)	
	21st day	42nd day	21st day	42nd day
Control	149.99 ± 34.33^c^	217.02 ± 68.32^c^	1.30 ± 0.55^a^	1.27 ± 0.08^b^
Bio-Mos	532.76 ± 33.16^a^	373.23 ± 14.54^b^	1.39 ± 0.20^a^	1.86 ± 0.06^ab^
*β*-Glucan	314.54 ± 8.67^b^	314.54 ± 8.67^bc^	1.54 ± 0.25^a^	1.77 ± 0.12^b^
DPS2	253.43 ± 56.19^ab^	490.45 ± 36.16^a^	1.79 ± 0.52^a^	2.62 ± 0.47^a^
DPS4	238.19 ± 42.08^ab^	431.70 ± 10.50^ab^	1.61 ± 0.28^a^	1.48 ± 2.20^b^
DPS6	437.39 ± 47.68^ab^	448.23 ± 4.98^ab^	1.78 ± 0.33^a^	1.26 ± 0.17^bc^

Means within the same column carrying different letters are significantly different at *P* < 0.05.

Values are expressed as means ± SE.
